# The U-Shape Relationship Between Glycated Hemoglobin Level and Long-Term All-Cause Mortality Among Patients With Coronary Artery Disease

**DOI:** 10.3389/fcvm.2021.632704

**Published:** 2021-02-26

**Authors:** Liwei Liu, Jianfeng Ye, Ming Ying, Qiang Li, Shiqun Chen, Bo Wang, Yihang Lin, Guanzhong Chen, Zhubin Lun, Haozhang Huang, Huangqiang Li, Danyuan Xu, Ning Tan, Jiyan Chen, Jin Liu, Yong Liu

**Affiliations:** ^1^Guangdong Provincial Key Laboratory of Coronary Heart Disease Prevention, Department of Cardiology, Guangdong Cardiovascular Institute, Guangdong Provincial People's Hospital, Guangdong Academy of Medical Sciences, Guangzhou, China; ^2^The Second School of Clinical Medicine, Southern Medical University, Guangzhou, China; ^3^Department of Cardiology, Dongguan Traditional Chinese Medicine Hospital, Dongguan, China; ^4^School of Medicine, Guangdong Provincial People's Hospital, South China University of Technology, Guangzhou, China

**Keywords:** coronary artery disease, glycated hemoglobin, all-cause mortality, U-shape, optimal

## Abstract

**Background:** Although glycated hemoglobin (HbA1c) was considered as a prognostic factor in some subgroup of coronary artery disease (CAD), the specific relationship between HbA1c and the long-term all-cause death remains controversial in patients with CAD.

**Methods:** The study enrolled 37,596 CAD patients and measured HbAlc at admission in Guangdong Provincial People's Hospital. The patients were divided into 4 groups according to HbAlc level (Quartile 1: HbA1c ≤ 5.7%; Quartile 2: 5.7% < HbA1c ≤ 6.1%; Quartile 3: 6.1% < HbA1c ≤ 6.7%; Quartile 4: HbA1c > 6.7%). The study endpoint was all-cause death. The restricted cubic splines and cox proportional hazards models were used to investigate the association between baseline HbAlc levels and long-term all-cause mortality.

**Results:** The median follow-up was 4 years. The cox proportional hazards models revealed that HbAlc is an independent risk factor in the long-term all-cause mortality. We also found an approximate U-shape association between HbA1c and the risk of mortality, including increased risk of mortality when HbA1c ≤ 5.7% and HbA1c > 6.7% [Compared with Quartile 2, Quartile 1 (HbA1c ≤ 5.7), aHR = 1.13, 95% CI:1.01–1.26, *P* < 0.05; Quartile 3 (6.1% < HbA1c ≤ 6.7%), aHR = 1.04, 95% CI:0.93–1.17, *P* =0.49; Quartile 4 (HbA1c > 6.7%), aHR = 1.32, 95% CI:1.19–1.47, *P* < 0.05].

**Conclusions:** Our study indicated a U-shape relationship between HbA1c and long-term all-cause mortality in CAD patients.

## Introduction

Glycated hemoglobin (HbA1c), as a biomarker that reflects 2–3 months of blood glucose status, plays a pivotal role in microvascular disease and atherosclerosis (1, 2). Previous studies have shown that HbA1c was associated with cardiovascular events and all-cause mortality (3, 4).

In recent years, abnormal glucose metabolism in patients with coronary artery disease (CAD) have received widespread attention. A higher level of baseline HbA1c was an independent predictor for poor prognosis in patients with acute myocardial infarction (AMI) or stable CAD (5, 6). However, the relationship between HbA1c levels and the long-term prognosis in CAD patients is not well-defined and some studies have reported conflicting results (7). She et al. found that AMI patients with different HbA1c levels showed no difference in prognosis during 2-year follow-up (8). A meta has found that there is no significant relationship between HbA1c levels and all-cause mortality among diabetic patients after percutaneous coronary intervention (PCI) (9). Besides, recent studies have found that there is not a simple linear relationship between HbA1c and all-cause mortality (6, 10).

Therefore, we aimed to investigate the specific association between baseline HbA1c and long-term all-cause mortality in CAD patients, as well as the optimal range for the lowest risk of mortality.

## Methods

### Study Design and Participants

This was a single-center, observational prospective study, which was completed in Guangdong Provincial People's Hospital (ClinicalTrials.gov NCT04407936). A total of 88,938 patients underwent coronary angiography from January 2008 to December 2018. 59,667 patients with a final diagnosis of CAD according to the 10th Revision Codes of the International Classification of Diseases (ICD-10; I20.xx–I25.xx, I50.00001 and I91.40001). A total of 37,596 CAD patients were included in the final analysis after excluding patients who lacked off HbAlc examination or follow-up information (Supplementary Figure 1). This research program was performed according to the Declaration of Helsinki and approved by The Ethics Committee of Guangdong Provincial People's Hospital. Baseline information such as demographic characteristics, clinical settings, laboratory examinations and medications at discharge were extracted from the electronic Clinical Management System of the Guangdong Provincial People's Hospital. Clinicians assessed whether the patients were feasible for Coronary angiography (CAG) or PCI based on the patient's condition and standard clinical practice guidelines (11).

### HbAlc Measurement

The serum HbAlc was measured by an ion-exchange high performance liquid chromatography with Bio-Rad Variant Testing System (Bio-Rad Laboratories, USA) at the time of admission.

### Definition and Endpoint

We calculated the estimated glomerular filtration rate (eGFR) by applying the Modification of Diet in Renal Disease (MDRD) equation (12). Diabetes mellitus (DM), and hypertension were defined using the ICD-10 code (Supplementary Table 1). The primary endpoint of this study was long-term all-cause death, incident events were defined as the first event occurring between the date of enrollment and the end of follow-up of December 31, 2018. Trained nurses monitored and recorded follow-up data through outpatient interviews and telephones.

### Statistical Analysis

Statistical analysis for this study was performed from January 1, 2008 to December 31, 2018. We divided the patients into 4 groups according to the quartile of serum HbAlc level (Quartile 1: HbA1c ≤ 5.7; Quartile 2: 5.7 < HbA1c ≤ 6.1; Quartile 3: 6.1 < HbA1c ≤ 6.7; Quartile 4: HbA1c > 6.7). We reported descriptive statistics by means (SD), median [interquartile range (IQR)], or number and percentage when appropriate. We used one-way analysis of variance (ANOVA) when analyzing differences between different groups. When analyzing categorical data, we used the Pearson chi-squared test. Prognosis analysis was used by Kaplan–Meier methods and survival curves. Log-rank test to compare the survival differences between the four groups of patients.

We used cox proportional hazards regression models and restricted cubic splines to evaluate the relationship between serum HbAlc levels and all-cause mortality in CAD patients. Hazard ratios and 95% CIs are reported. Model 1 was adjusted with age > 75 years and gender, and model 2 was adjusted eGFR. Model 3, as the primary results, was adjusted with the variables which were significant at *P* < 0.05 according to univariate Cox proportional hazards regression and associated with mortality according to clinical experience (included history of present illness information, drugs information). The proportional hazards assumption was tested with the use of Schoenfeld residuals. Variables with missing values > 30% were not considered as candidates. We conducted subgroup analysis based on patients' characteristics and comorbidities stratified by age, gender, diabetes, AMI, PCI, renal insufficiency (eGFR <60). All data analyses were performed using R (version 3.6.3; R Core Team, Vienna, Austria). *P*-values < 0.05 were considered to represent statistical significance.

## Result

### Clinical Characteristics

We included 37,596 patients in the final analyses. Baseline clinical of the study patients are shown in Table 1. The mean age was 62.54 ± 10.55 years, and 27,007 (71.83%) were male. The distribution of HbA1c as follows: mean, 6.43 ± 1.33 %. Patients were divided into four groups: Quartile 1 (HbA1c ≤ 5.7, *n* = 11,094), Quartile 2 (5.7 < HbA1c ≤ 6.1, *n* = 9,684), Quartile 3 (6.1 < HbA1c ≤ 6.7, *n* = 7,823), Quartile 4 (HbA1c > 6.7, *n* = 8,995). Nine thousand four hundred forty-three (27.45%) patients complicated with DM and 19,224 (55.88%) patients complicated with hypertension. Five thousand sixty-three (14.72%) patients identified in AMI and 19,760 (52.56%) patients treated with PCI (Table 1).

**Table 1:** Baseline characteristics of the patients.

**Characteristic**		**HbA1c level Quartile**				
	**Overall** **(*n* = 37,596)**	**≤5.7%** **(*n* = 11,094)**	**5.7–6.1%** **(*n* = 9,684)**	**6.1–6.7%** **(*n* = 7,823)**	**>6.7%** **(*n* = 8,995)**	***p*-value**
**Demographic**						
Age, year	62.54 (10.55)	60.39 (11.26)	62.86 (10.34)	64.19 (10.01)	63.43 (9.88)	<0.001
Age > 75, *n* (%)	5,150 (13.70)	1,376 (14.21)	1,220 (11.00)	1,285 (16.43)	1,269 (14.11)	<0.001
Male, *n* (%)	27,007 (71.83)	8,249 (74.36)	7,035 (72.65)	5,589 (71.44)	6,134 (68.19)	<0.001
**Medical history**						
AMI, *n* (%)	5,063 (14.72)	1,551 (14.91)	1,284 (14.53)	913 (12.94)	1,315 (16.22)	<0.001
Pre-MI, *n* (%)	2,043 (5.94)	500 (5.66)	526 (5.06)	453 (6.42)	564 (6.96)	<0.001
Diabetes, *n* (%)	9,443 (27.45)	640 (7.24)	400 (3.84)	1,717 (24.34)	6,686 (82.46)	<0.001
Hypertension, *n* (%)	19,224 (55.88)	4,971 (47.78)	4,704 (53.25)	4,281 (60.69)	5,268 (64.97)	<0.001
PCI, *n* (%)	19,760 (52.56)	5,151 (46.43)	4,951 (51.13)	4,184 (53.48)	5,474 (60.86)	<0.001
**Laboratory test**						
eGFR, ml/min/1.73 m^2^	80.84 (25.62)	82.25 (25.55)	83.69 (23.77)	78.17 (24.84)	78.30 (27.85)	<0.001
WBC, 109/L	7.63 (2.42)	7.37 (2.38)	7.52 (2.38)	7.69 (2.38)	8.00 (2.50)	<0.001
HGB, g/L	133.14 (16.61)	134.24 (16.64)	134.12 (15.77)	132.61 (16.37)	131.20 (17.43)	<0.001
CHOL, mmol/L	4.50 (1.18)	4.46 (1.14)	4.53 (1.14)	4.53 (1.20)	4.50 (1.24)	<0.001
TRIG, mmol/L	1.65 (1.20)	1.48 (0.96)	1.56 (1.06)	1.68 (1.16)	1.92 (1.53)	<0.001
APOB, g/L	0.86 (0.24)	0.84 (0.23)	0.86 (0.23)	0.86 (0.24)	0.87 (0.25)	<0.001
LDLC, mmol/L	2.80 (0.95)	2.79 (0.94)	2.82 (0.95)	2.82 (0.97)	2.77 (0.97)	<0.001
HbA1c, %	6.43 (1.33)	5.39 (0.34)	5.94 (0.11)	6.40 (0.17)	8.27 (1.50)	<0.001
URIC, μmol/L	396.06 (112.37)	387.06 (107.36)	396.52 (108.09)	411.50 (112.87)	393.40 (120.94)	<0.001
ALB, g/L	36.95 (4.13)	37.46 (4.02)	36.99 (3.95)	36.79 (4.10)	36.42 (4.42)	<0.001
**Medication**						
ACEI/ARB, *n* (%)	23,825 (65.53)	6,255 (59.23)	6,002 (64.04)	5,249 (68.86)	6,319 (71.79)	<0.001
Beta-blockers, *n* (%)	23,008 (63.19)	6,420 (60.76)	5,797 (61.77)	4,899 (64.17)	5,892 (66.78)	<0.001
Statin, *n* (%)	32,717 (89.86)	9,200 (87.07)	8,458 (90.12)	6,910 (90.52)	8,149 (92.36)	<0.001
Death, *n* (%)	4,340 (11.54)	1,084 (9.77)	1,018 (10.51)	956 (12.22)	1,282 (14.25)	<0.001

*AMI, acute myocardial infarction; PCI, percutaneous coronary intervention; eGFR, estimated glomerular filtration rate; WBC, white blood cell; HGB, hemoglobin; CHO, serum total cholesterol; TRIG, triglyceride; APOB, apolipoprotein B; LDL-C, low-density lipoprotein cholesterol; HbA1c, hemoglobin A1c; URIC, uric acid; ALB, albumin; ACEI/ARB, angiotensin-converting enzyme inhibitor/angiotensin receptor blocker*.

### Main Outcomes

**Figure 1:**
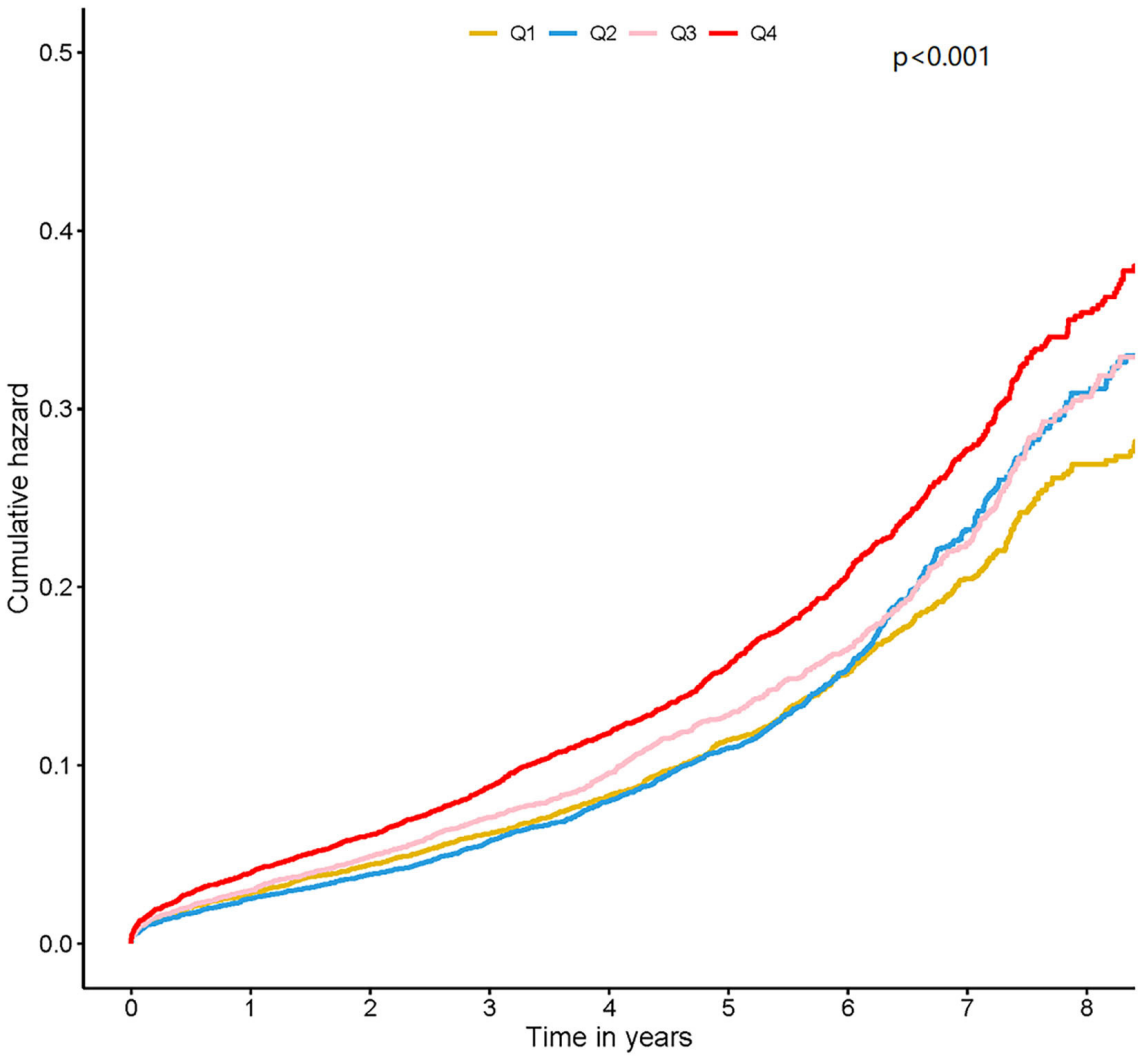
Kaplan-Meier curves for quartile values of plasma levels of HbA1c. Quartile 1: HbA1c ≤ 5.7; Quartile 2: 5.7 < HbA1c ≤ 6.1; Quartile 3: 6.1 < HbA1c ≤ 6.7; Quartile 4: HbA1c > 6.7.

The median follow-up period was 4.0 (2.2–5.9) years and prognostic data were fully documented during the entire follow-up period. Four thousand three hundred forty (11.54%) patients died during the follow-up. Kaplan–Meier curves analysis revealed that patients in the highest HbA1c level group (Quartile 4) had a significantly higher long-term mortality compared with those in the lower HbA1c group (Quartile1 or 2 or 3) (log-rank analysis *P* < 0.01) (Figure 1).

**Figure 2:**
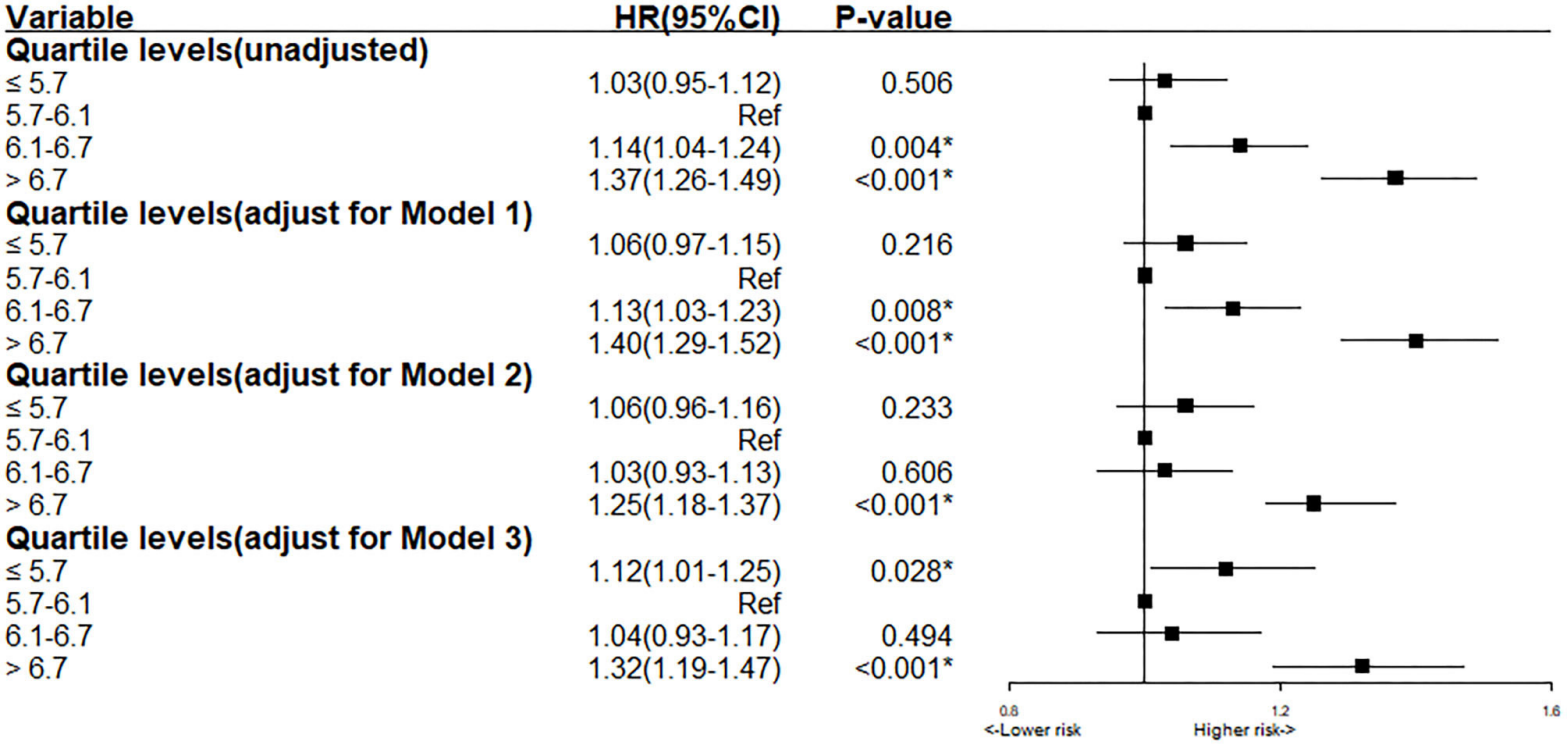
Cox proportional hazard ratios for long-term all-cause mortality in different models. Model 1: Cox proportional hazard ratio for long-term all-cause mortality adjusted for age > 75 and gender. Model 2: Cox proportional hazard ratio for long-term all-cause mortality adjusted for eGFR. Model 3: Cox proportional hazard ratio for long-term all-cause mortality adjusted for multiple variables (age > 75, gender, AMI, HT, pre-MI, PCI, eGFR, LDLC, URIC, Statin, ACEI/ARB).

**Figure 3:**
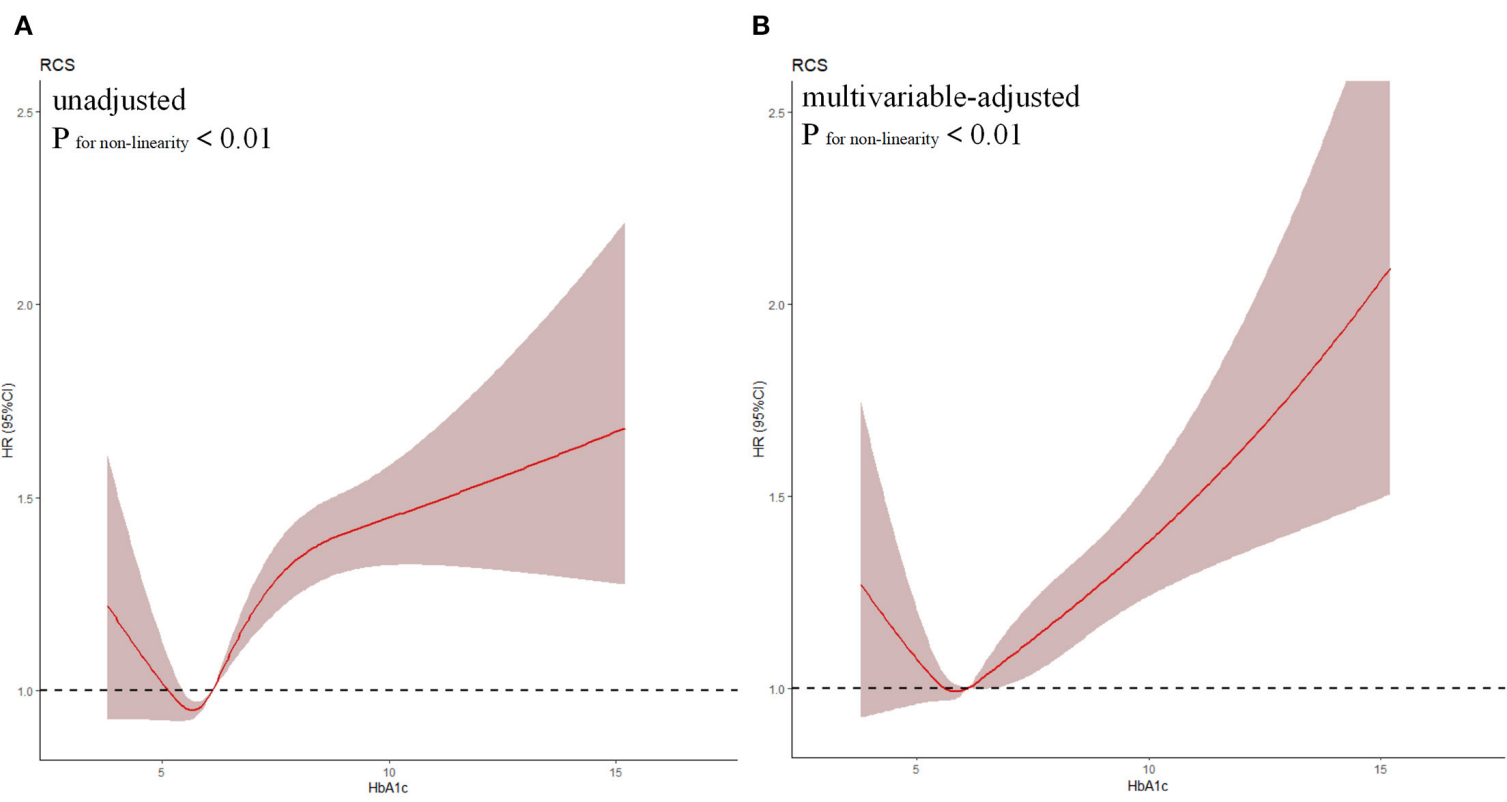
Restricted spline curve of the HbA1c hazard ratio for mortality. **(A)** The restrict spline curve of univariate cox model; **(B)** The restrict spline curve of multivariate cox model, Adjusted for age > 75, gender, AMI, HT, pre-MI, PCI, eGFR, LDLC, URIC, Statin, ACEI/ARB.

Based on the result of the univariate regression analysis, 10 variables (including gender, hypertension, AMI et al.) were significantly associated with the long-term all-cause mortality (Supplementary Table 2). In Cox analysis, Quartile 2 (5.7 < HbA1c ≤ 6.1%) was served as the reference group. The results of Cox proportional hazards regression were shown in Figure 2. In Cox multivariate analysis, patients in the lowest or highest HbA1c level (Quartile 1 or 4) showed a significantly higher risk of long-term mortality than other groups (Quartile 2 or 3) [Compared with Quartile 2, Quartile 1 (HbA1c ≤ 5.7), aHR = 1.13, 95% CI:1.01–1.26, *P* < 0.05; Quartile 3 (6.1 < HbA1c ≤ 6.7%), aHR = 1.04, 95% CI:0.93–1.17, *P* = 0.49; Quartile 4 (HbA1c > 6.7%), aHR = 1.32, 95% CI:1.19–1.47, *P* < 0.05]. The relationship between HbA1c and all-cause mortality was non-linear (non-linear *P* < 0.001). In the univariate and multivariate cox models of restricted cubic splines, we observed a U-shaped association between HbA1c and long-term all-cause mortality (Figure 3); low and high levels of HbA1c were associated with an increased risk of all-cause mortality. The Cox models fulfilled the proportional-hazards assumption tested by Schoenfeld residual (*P* > 0.05).

### Subgroup Analysis

**Figure 4:**
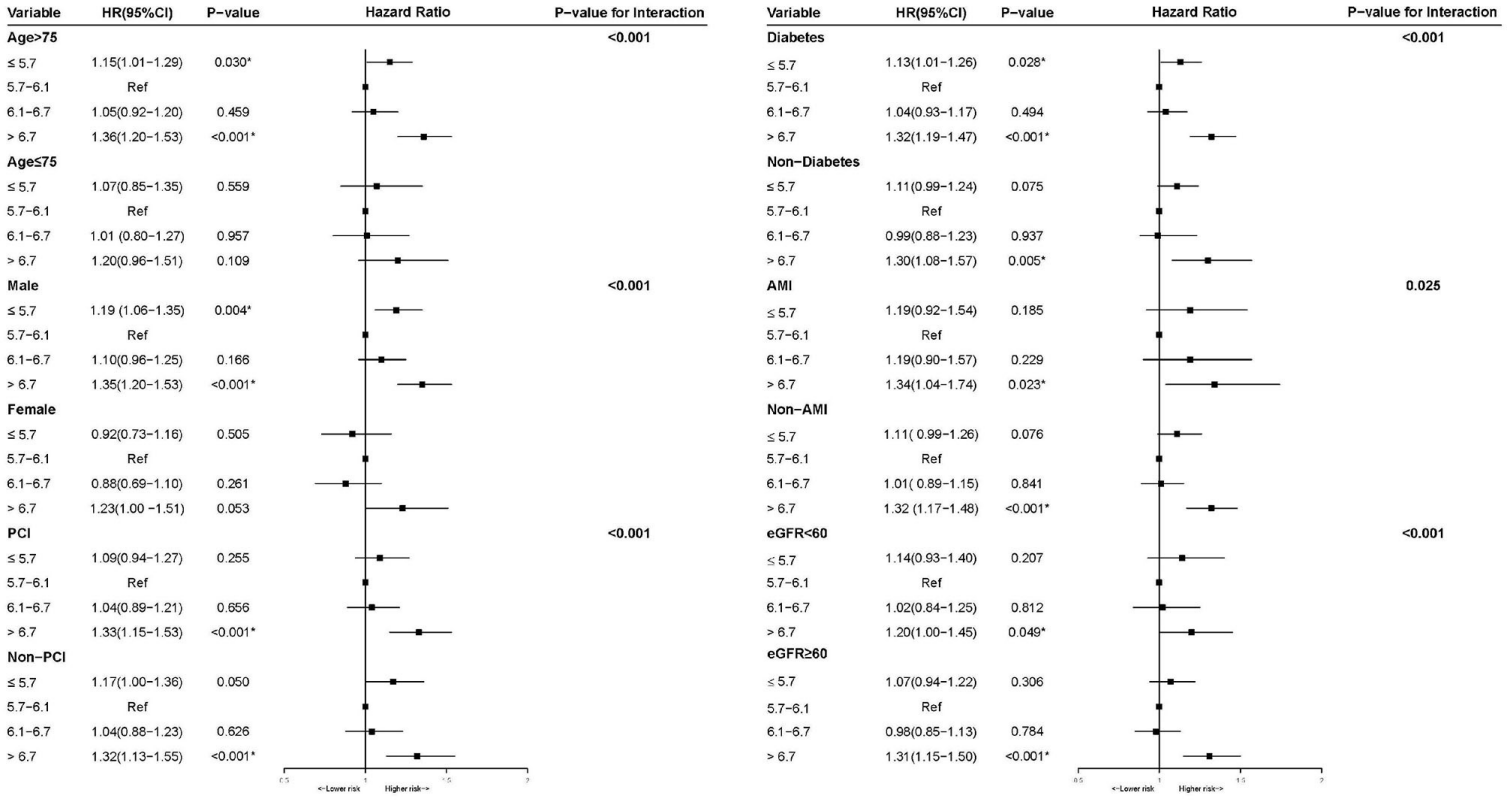
Multivariable cox proportional hazard ratios for long-term all-cause mortality in Subgroups stratified by patients' characteristics and comorbidities.

To assess whether the association between HbA1c and long-term all-cause mortality could be explained by patients' characteristics and comorbidities. We conducted multivariable-adjusted hazard ratios for all-cause mortality stratified by age, gender, PCI, diabetes, AMI and renal insufficiency (eGFR <60) (*P* for interaction <0.05). We observed the stable U-shape association between HbA1c and long-term mortality in most subgroups (Figure 4), although the association was slightly reduced in female patients (Figure 4). As shown in restricted cubic splines (Supplementary Figure 2), patients in the different subgroups showed U-shape association between HbA1c and long-term mortality.

## Discussion

To our knowledge, this is the largest cohort study of the value of HbA1c in CAD patients. In this study of 37,596 CAD patients with more 4 years follow-up, we found a U-shaped relationship between levels of HbA1c and the risk of all-cause mortality, with low and high levels associated with an increased risk.

The impact of abnormal glucose metabolism among patients with cardiovascular disease has drawn widespread attention (13–15). Previous studies have suggested that HbA1c is an independent risk factor for prognosis in CAD patients of different subgroups. Hong et al. found that elevated HbA1c would aggravate the severity of CAD and poor prognosis (6). Hamdi et al. demonstrated that increased HbA1c levels at admission were associated with higher rates of major adverse cardiovascular events and mortality in patients undergoing PCI (12). However, there is also some controversial finding (16, 17). She et al. found that AMI patients with different HbA1c levels showed no difference in prognosis during 2-year follow-up (8). Similarly, Lemesle et al. showed no significant effect of HbA1c on 1-year rate of major adverse cardiovascular events (including death) among 952 diabetic patients undergoing PCI (7). Most previous studies included limited number of patients with a relatively short follow-up, which may lead to controversial results. There is still a lack of discussion in large data of the CAD patients. In our research, we included large samples (more than 30,000) of CAD patients and found that HbA1c is an independent prognostic factor of mortality during more than 4 years of follow-up. Interestingly, we firstly found that the relationship between HbA1c and all-cause mortality was more similar to a U-shape than a linear association. Extremely low HbA1c level also leads to poor prognosis among patients with CAD.

In subgroup analysis, the U-shaped association between levels of HbA1c and long-term mortality in CAD patients is stable in most subgroups. However, the U-shape association was strong in male subgroup while slightly reduced in female subgroup. Previous studies have found that there were sex differences in the association of HbA1c and cardiovascular disease risk (18–20). The underlying mechanisms of our finding may be related to gender differences.

According to our findings, patients with excessively high HbA1c levels need to be reduced. As a new diabetes medication, sodium-glucose cotransporter-2 (SGLT2) inhibitors has been proved to reduce the risk of cardiovascular events and mortality by the reduction of HbA1c (21). Although the guideline recommends that some populations should consider less-rigorous targets of HbA1c levels to prevent complications (including hypoglycaemic episodes), there is no clear and appropriate range for the CAD population (21). Our study seems to discover a “security zone” of HbA1c levels for management among CAD patients based on a large sample. In the future, more studies should explore whether effective HbA1c control can improve the prognosis in the CAD population.

Previous researches suggested a correlation between plasma HbA1c and inflammatory biomarkers, chemical parameters, either alone or combined. These markers or risk factors had a direct role in the progression of atherosclerotic artery disease and adverse cardiovascular events (22–24). The increases in serum biomarkers of inflammation might further promote the development of secondary cardiac arrhythmias and heart failure (25). Meanwhile, hypoglycemia induces several changes in the hemostatic parameters which include an increase in platelet aggregation, activation, degranulation as well as a rise in factor VIII and vWF levels (26, 27). These effects are exaggerated in patients and are likely to be detrimental to myocardial circulation particularly in patients with an already compromised myocardial perfusion.

## Limitation

In this study, there are still several limitations. At first, this observational study was conducted in a single center. However, our study was derived from a southern central hospital with more than 30,000 patients from the southern cities, which can represent the information of patients with CAD in southern China. Second, as an observational study, there are uncertain risk factors (such as lifestyle, malignancy) that may cause residual confounding effects on long-term mortality. But the results in our study have adjusted for common cardiovascular prognostic factors. Thirdly, DM was defined according to the ICD code at admission. Although there are some undetected DM patients that might include in our study, this real-world study can also reflect the current situation of DM diagnosis and treatment among patients with CAD in China. Finally, only the HbA1c level at admission was included in our study and the change of value has lacked. Due to the characteristics of glycated hemoglobin, it can reflect a patient's long-term blood glucose status.

## Conclusions

Our study firstly indicated a U-shape relationship between HbA1c and long-term all-cause mortality in CAD patients. For these patients, the optimal HbA1c level (5.7–6.7%) may have a better prognosis. Further study is required to explore whether effective HbA1c control can improve the prognosis in the CAD population.

## Data Availability Statement

The raw data supporting the conclusions of this article will be made available by the authors, without undue reservation.

## Ethics Statement

All procedures were performed in accordance with the ethical standards of the responsible committee on human experimentation (institutional and national) and with the Helsinki Declaration of 1975. This study was approved by the institutional ethics board of Guangdong Province Peoples' Hospital. All patients were eligible in this study.

## Author Contributions

YLiu, JL, LL, JY, and JC: research idea and study design. JL, MY, QL, LL, SC, BW, YLin, GC, HH, HL, NT, and ZL: data acquisition. JL and YLiu: data analysis/interpretation. DX and MY: statistical analysis. YLiu, JC, and NT: supervision and mentorship. All authors contributed important intellectual content during manuscript drafting or revision and accepts accountability for the overall work by ensuring that questions pertaining to the accuracy or integrity of any portion of the work are appropriately investigated and resolved.

## Conflict of Interest

The authors declare that the research was conducted in the absence of any commercial or financial relationships that could be construed as a potential conflict of interest.

## Supplementary Material

Supplementary Figure 1 The flow of participants through the trial.

Supplementary Figure 2 Restricted spline curve of the HbA1c hazard ratio for mortality in Subgroups stratified by patients' characteristics and comorbidities.

Supplementary Table 1 Univariable Cox regression analysis of long-term all-cause mortality.

Supplementary Table 2 The ICD-10 codes information of diagnoses.
