# Supplementary figures and images for: The U-Shape Relationship Between Glycated Hemoglobin Level and Long-Term All-Cause Mortality Among Patients With Coronary Artery Disease
Source: Front Cardiovasc Med. 2021 Feb 26;8:632704. doi: 10.3389/fcvm.2021.632704 (PMC7952311; doi:10.3389/fcvm.2021.632704)

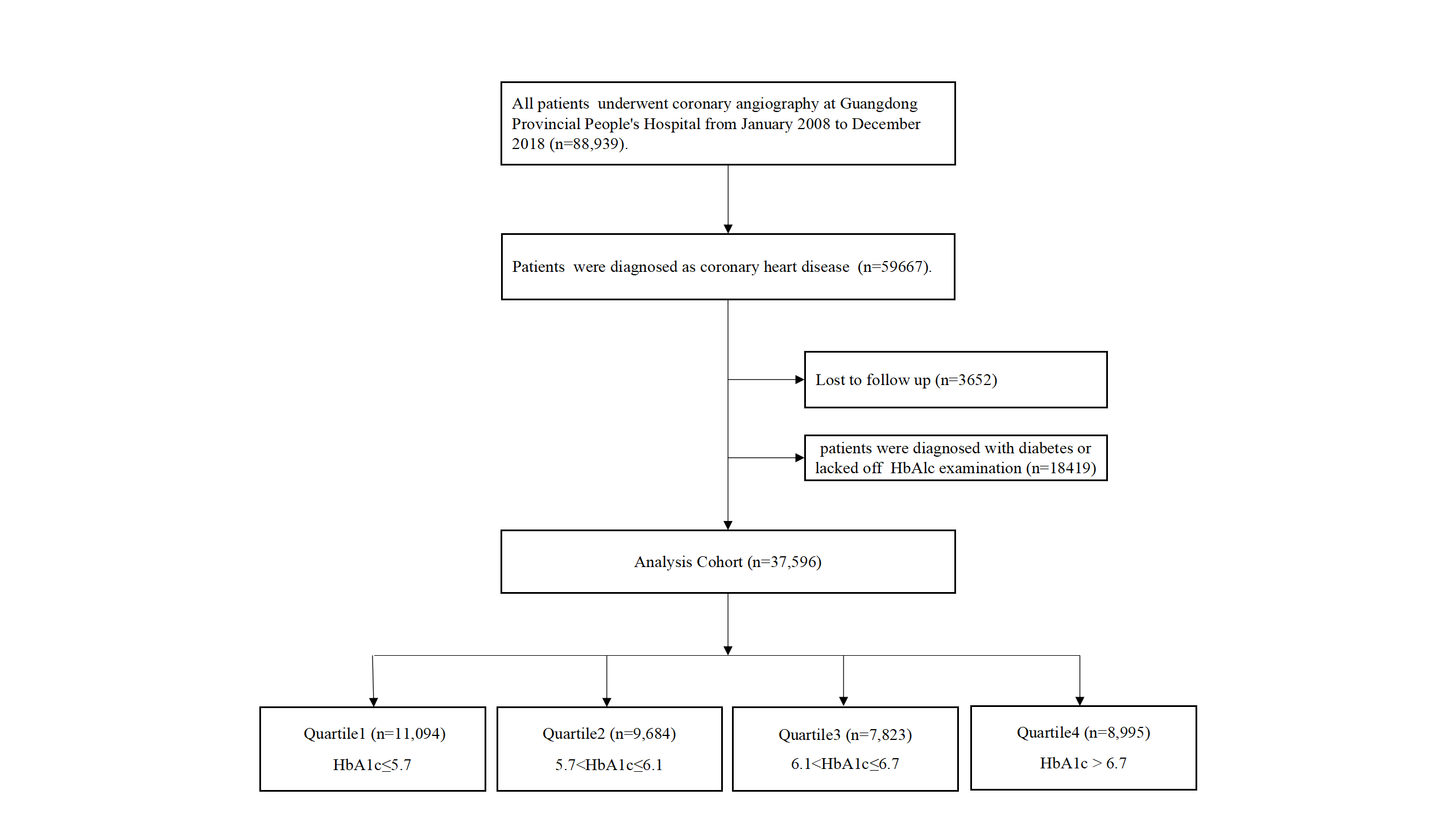

Supplement: Supplementary Figure 1 — The flow of participants through the trial. [file Image_1.PNG]

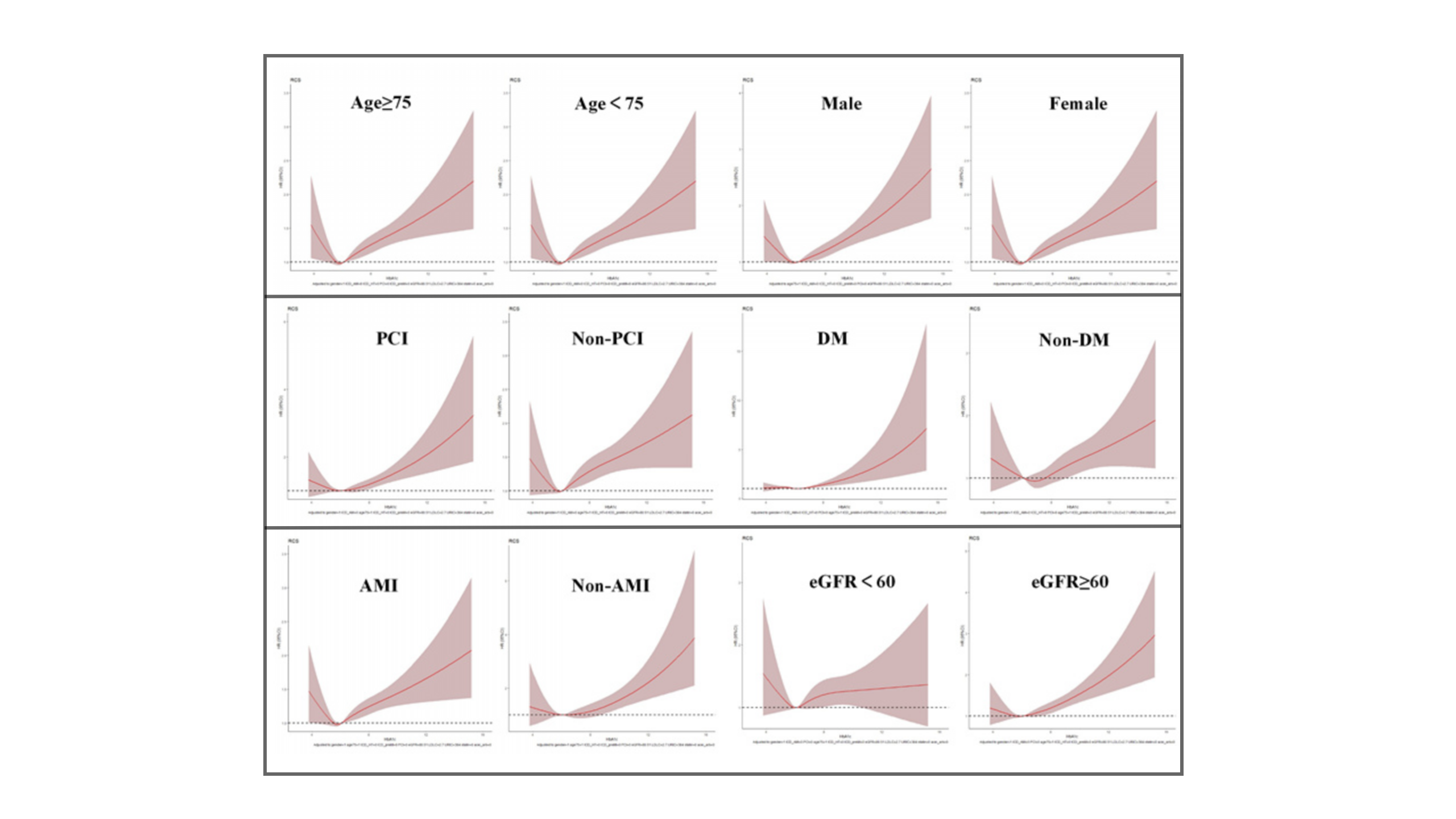

Supplement: Supplementary Figure 2 — Restricted spline curve of the HbA1c hazard ratio for mortality in Subgroups stratified by patients' characteristics and comorbidities. [file Image_2.PNG]
