# Supplementary material for: The U-Shape Relationship Between Glycated Hemoglobin Level and Long-Term All-Cause Mortality Among Patients With Coronary Artery Disease
Source: Front Cardiovasc Med. 2021 Feb 26;8:632704. doi: 10.3389/fcvm.2021.632704 (PMC7952311; doi:10.3389/fcvm.2021.632704)
Supplement: Supplementary Table 1 — Univariable Cox regression analysis of long-term all-cause mortality. [file Data_Sheet_1.docx]

**Supplemental Table S1. Univariable Cox regression analysis of long-term all-cause mortality.**

| **variable** | **Univariate** | |
| --- | --- | --- |
|  | HR（95% CI） | p value |
| Age | 1.03(1.03-1.03) | ＜0.001 |
| Age >75 | 1.98(1.84-2.12) | ＜0.001 |
| Male | 0.85(0.79-0.91) | ＜0.001 |
| AMI | 1.20(1.11-1.30) | ＜0.001 |
| Hypertension | 1.13(1.06-1.20) | ＜0.001 |
| Pre-MI | 1.20(1.08-1.34) | ＜0.001 |
| PCI | 0.87(0.82-0.92) | ＜0.001 |
| APOB | 1.10(0.96-1.27) | 0.158 |
| LDLC | 0.98(0.95-1.01) | 0.158 |
| URIC | 1.00(1.00-1.00) | ＜0.001 |
| eGFR | 0.98(0.98-0.98) | ＜0.001 |
| ACEI/ARB | 0.89(0.84-0.95) | ＜0.001 |
| Beta-blockers | 0.94(0.88-1.00) | 0.064 |
| Statin | 0.86(0.77-0.95) | ＜0.003 |

AMI: acute myocardial infarction. PCI: percutaneous coronary intervention. APOB: apolipoprotein b. LDL-C: Low-density Lipoprotein cholesterol. URIC: uric acid. eGFR: estimated glomerular filtration rate. ACEI/ARB: angiotensin-converting enzyme inhibitor/angiotensin receptor blocker.

**Supplemental Table S2. The ICD-10 codes information of diagnoses.**

| Diagnosis | ICD-10 codes |
| --- | --- |
| Coronary Artery disease | CD-10; I20.xx–I25.xx, I50.00001 and I91.40001 |
| Hypertension | I10.x00, I10.x05, I10.x04, I10.x03, I11.900, I10. 13, I12.900, H35.004, I12.903, I15.900, I67.400, I13.900, I15.800, I11.000, I12.000, I10.x06, I10.x01, I13.100, I10.x04, I10. 03, I10.x05, I10.x03, R03.000, I11.901, I10.x09, I10.x07, I10.x00, I15.102, I12.900, I11.900, I11.002, I12.000, I10.x11, I13.900, I15.103, I15.200, I11.000, I11.001, I10.x12, I10.x10, I13.000, |
| Diabete mellitus | E11.900, E14.900, E11.901, E11.300, E11.301+H36.0, E11.401+G63.2, E11.501+I79.2, E11.601, E11.700, E11.200+N08.3, E10.900, E13.905, E11.800, E13.903, E11.400, E11.200+N08.3, E13.300+H36.0, E13.200+N08.3, E11.100, E13.907, E11.10001, E11.60001, E14.10001, E11.500, E10.401+G63.2, E11.403+G63.2, E13.400+G63.2, E13.500, E13.700, Z83.300, E11.502, E11.60002, E10.201, E11.901, N08.301*, E11.900, E11.700, E11.200, E11.90002, E11.201+N08.3, E14.900, E13.900, E14.300, E14.800, E13.200, E11.800, O24.300, E14.200, O24.100, E13.800, E11.503, E11.400, E13.600, E14.600, E11.302+H28.0, E14.500, E14.400, E11.500, E11.101, E13.201+N08.3, E11.70001, E14.700, E11.502+I79.2, E13.400, E11.100, E11.103, E11.300, E13.300, E10.400, E13.500, E11.505, E12.000, E11.600, E13.700, E11.604, E11.402+G99.0, R73.003 |
